# CD169 ^+^ sinus macrophages in regional lymph nodes do not predict mismatch‐repair status of patients with colorectal cancer

**DOI:** 10.1002/cam4.5747

**Published:** 2023-02-27

**Authors:** Yoichi Saito, Yukio Fujiwara, Yuji Miyamoto, Koji Ohnishi, Yuta Nakashima, Yasuhiko Tabata, Hideo Baba, Yoshihiro Komohara

**Affiliations:** ^1^ Department of Cell Pathology, Graduate School of Medical Sciences, Faculty of Life Sciences Kumamoto University Kumamoto Japan; ^2^ Laboratory of Biomaterials Institute for Frontier Life and Medical Sciences, Kyoto University Kyoto Japan; ^3^ Laboratory of Bioengineering, Faculty of Advanced Science and Technology Kumamoto University Kumamoto Japan; ^4^ Japan Society for the Promotion of Science Tokyo Japan; ^5^ Department of Gastroenterological Surgery, Graduate School of Medical Sciences, Faculty of Life Sciences Kumamoto University Kumamoto Japan; ^6^ Institute of Industrial Nanomaterials, Kumamoto University Kumamoto Japan; ^7^ International Research Organization for Advanced Science and Technology Kumamoto University Kumamoto Japan; ^8^ Fusion Oriented Research for Disruptive Science and Technology Japan Science and Technology Agency Saitama Japan

**Keywords:** CD169, colorectal cancer, dMMR/MSI‐H, PD‐L1, regional lymph node, sinus macrophage, tumor‐infiltrating T cells

## Abstract

**Aims:**

Mismatch‐repair deficiency and microsatellite instability‐high (dMMR/MSI‐H) colorectal cancer (CRC) is treated with programmed death (PD)‐1 antibody regardless of PD‐ligand (L)1 expression in tumor cells. We previously found that abundant CD169^+^ macrophages in regional lymph node (RLN) sinuses and CD8^+^ tumor‐infiltrating lymphocytes (TILs) positively correlated in CRC and were associated with a favorable prognosis. However, associations between dMMR/MSI‐H CRC and CD8^+^ TILs or prognoses vary among studies. In this study, we attempted to compare the association between MMR status, CD169^+^ macrophages in RLNs, CD8^+^ TILs, PD‐L1 scores, and prognoses in CRC.

**Methods and Results:**

We immunostained 83 surgically resected CRC tumors that we previously analyzed for MMR proteins, and identified 9 that were dMMR. The number of CD169^+^ macrophages in RLNs and CD8^+^ TILs significantly correlated with overall survival, whereas MMR status did not. The number of cells positive for the TIL markers CD3, CD4, CD8, and TIA‐1, and macrophage markers CD68 and CD169 in RLNs did not significantly differ between groups according to MMR status. Furthermore, combined positive scores (CPS) for PD‐L1 expression in five of nine dMMR CRCs were all <1. We found that dMMR in CRC did not correlate with numbers of CD169^+^ macrophages in RLNs or CD8^+^ TILs.

**Conclusions:**

CRC with CD169^+^ macrophages in RLNs and abundant CD8^+^ TILs indicates a better prognosis and it should be immunologically classified as a different antitumor group from dMMR CRC.

## Graphical abstract

Colorectal cancer with high number of CD169^+^ macrophages in regional lymph nodes indicates a better prognosis independently of MMR status. Furthermore, dMMR should be immunologically classified into a different antitumor group from colorectal cancers with abundant CD8^+^ tumor‐infiltrating T cells.

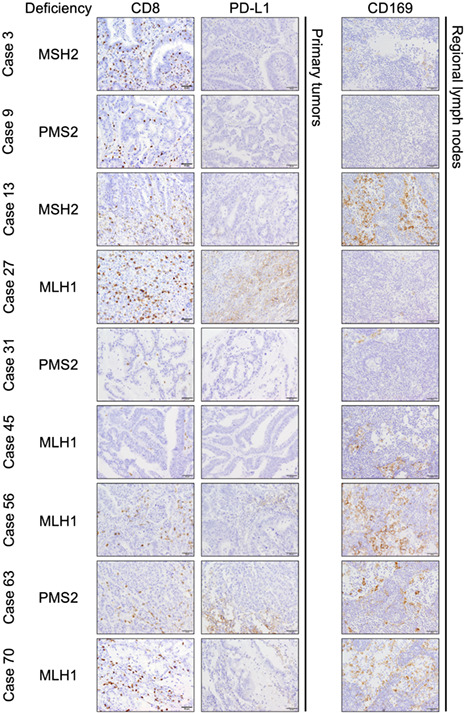

## 1 INTRODUCTION

Colorectal cancer (CRC) is a common, fatal type of cancer worldwide. The World Health Organization (WHO) has reported that CRC is the third and second most prevalent type of cancer in men and women, respectively (https://gco.iarc.fr). The usual treatment for all stages of CRC comprises surgery, followed by chemotherapy, immunotherapy, and radiotherapy as appropriate. However, CRC remains the fourth and fifth most common causes of cancer‐related death among men and women, respectively. Moreover, the prevalence of CRC is likely to increase in the near future.^1^

Mismatch‐repair deficiency and microsatellite instability‐high (dMMR/MSI‐H) subsets contain numerous tumor mutations. The size of nucleotide repeat sequences (microsatellites) is altered in dMMR/MSI‐H CRC due to mutations or the inactivation of any one of the MMR genes: PMS2, MSH6, MLH1, and MSH2.^2^, ^3^, ^4^ Such tumors generate more neoantigens than those that are mismatch‐repair proficient and microsatellite stable (pMMR/MSS), which explains the firm priming of T‐cell‐mediated adaptive antitumor immunity.^5^, ^6^, ^7^, ^8^ In fact, more tumor‐infiltrating lymphocytes (TILs) have been found in MSI‐H than in MSS CRC.^9^ Furthermore, dMMR/MSI‐H is considered to indicate a more optimistic prognosis for patients with untreated stage II CRC.^10^ However, a recent meta‐analysis of stage III and IV CRC did not identify a clear correlation between dMMR/MSI‐H and good prognosis.^11^ That is, whether dMMR/MSI‐H is a favorable prognostic factor remains unclear.

The simplest (and recommended) method for detecting dMMR is to immunohistochemically stain MMR proteins.^12^ These proteins will not be immunostained if MMR genes are mutated or inactivated. The MMR proteins MLH1 and MSH2 form functional heterodimers with PMS2 and MSH6, respectively.^13^, ^14^ Thus, immunohistochemical (IHC) staining of PMS2 and MSH6 can also detect mutations in MLH1 and MSH2, respectively. Therefore, an antibody panel that includes PMS2 and MSH6 is sufficient to screen for dMMR.^15^

The immune checkpoint inhibitors (ICIs), programmed death receptor‐1 (PD‐1)/programmed death‐ligand 1 (PD‐L1) antibodies are effective against many types of cancer.^16^, ^17^, ^18^, ^19^, ^20^ PD‐L1 is mainly expressed in tumor cells and is positively regulated by IFN‐γ secreted from CD8^+^ TILs.^21^, ^22^, ^23^ The antibodies block binding PD‐1 in CD8^+^ T cells and PD‐L1 in tumor cells or some immune cells, preventing immune tolerance and tumor progression.^24^ Considering this concept, PD‐L1 is immunohistochemically assessed as a companion diagnostic test (CDx) before administering pembrolizumab to patients with some types of cancer.^25^, ^26^, ^27^, ^28^, ^29^ Pembrolizumab and nivolumab have been applied to treat dMMR/MSI‐H CRC rather than CRC with abundant PD‐L1 expression.^30^, ^31^, ^32^ The expression of PD‐L1 is not likely to correlate with dMMR/MSI‐H status in CRC.^12^ This means that dMMR/MSI‐H and high PD‐L1 are not simply connected by T‐cell‐mediated adaptive antitumor immunity.

Regional lymph nodes (RLNs) are primary sites of the immune response to tumor immunity. Dead tumor cells or fragments flow via lymph vessels into the sinus areas of RLNs, where they are endocytosed by sinus macrophages.^33^ These macrophages internalize, process, present tumor antigens on MHC I, and activate tumor antigen‐specific lymphocytes, especially CD8^+^ T cells.^34^, ^35^ These findings suggest that sinus macrophages in RLNs are critical to the antitumor immune response.

The 185‐kDa transmembrane receptor, CD169 (also known as sialoadhesin or sialic acid‐binding lectin; Siglec 1), is expressed in LN sinus macrophages,^36^ and in macrophages induced by IFN‐α, −β, and ‐γ in vitro.^37^, ^38^ CD169 binds sialylated glycoproteins, including CD43 (sialophorin) and MUC1, and participates in intercellular adhesion or cell‐pathogen interactions.^36^ Dead tumor cell antigens are phagocytosed by CD169^+^ sinus macrophages in RLNs; then, the proliferation of antigen‐specific CD8^+^ T cells are induced via cross‐presentation in tumor vaccination and transplantation mouse models, resulting in tumor rejection.^39^ These findings indicate that CD169^+^ macrophages in RLNs are important to establish T‐cell‐mediated adaptive antitumor immunity.

Abundant CD169^+^ macrophages in RLNs are associated with a high density of tumor‐infiltrating CD8^+^ T or NK cells and a better clinical prognosis for patients with CRC and several other types of malignant tumors.^37^, ^38^, ^40^, ^41^, ^42^, ^43^, ^44^, ^45^ That is, CD169 expression in sinus macrophages can monitor CD8^+^ TILs and predict prognosis. However, CD169^+^ macrophages in RLNs and MMR status have never been directly compared in any type of cancer.

In the present study, we immunohistochemically investigated 83 samples of previously analyzed CRC and identified 9 with dMMR. We analyzed CD169^+^ macrophages in RLNs, and TIL, and PD‐L1 expression in primary tumors. We also statistically compared prognoses and correlations with individual parameters using updated clinical data.

## 2 MATERIALS AND METHODS

### 2.1 Patients

Specimens of primary tumors and RLN samples from 83 patients were formalin‐fixed and paraffin‐embedded^37^ (Table 1). All patients had undergone surgery to treat CRC between 2000 and 2006 at Kumamoto University Hospital. All patients provided written informed consent to participate in the study; the study protocols were approved by the Institutional Review Board at Kumamoto University Hospital (approval no. 1016).

**TABLE 1:** Patient characteristics.

Characteristic	*n* (total = 83)	%
Sex		
Female	35	42
Male	48	58
Age (years)		
Median (range)	64 (29–90)	
Mean	67.7	
Histologic subtype		
Well differentiated	49	59
Moderately differentiated	26	31
Poorly differentiated	1	1
Mucinous	6	7
Other	1	1
T stage (invasion depth)		
Tis	0	0
T1 (SM)	6	7
T2 (MP)	13	16
T3 (SS, A)	47	57
T4a (SE)	11	13
T4b (SI, AI)	6	7
N stage		
N0	54	65
N1	19	23
N2	9	11
N3	1	1
Stage		
I	17	20
II	32	39
III	18	22
IV	16	19

### 2.2 Immunostaining

Sections (3 μm) of CRC and RLN tissues were stored at −80°C. We immunostained PMS2 (clone EP51), MSH6 (clone EP49), MLH1 (clone ES05), MSH2 (clone FE11), CD8 (clone C8/144B), and PD‐L1 (clone 22C3) using EnVision FLEX, pharmDx Dako kit reagents, and Dako Autostainer Link 48 (Agilent Technologies Inc.). The sections were immersed in EDTA (pH 8.0) (CD3, CD68, and CD169) or antigen retrieval solution (pH 9.0) (Nichirei Bioscience Inc.) (CD4 and TIA‐1) and heated in a pressure cooker (CD3, CD68, and TIA‐1), microwave (CD169), or hot bath (CD4). The sections were then incubated with the primary antibodies: CD3 (clone SP7) and CD4 (clone 1F6) (both from Nichirei Biosciences Inc.), CD68 (clone PG‐M1; Agilent Technologies Inc.), CD169 (clone 7D2; Santa Cruz Biotechnology Inc.), and TIA‐1 (clone TIA‐1; Abcam). Thereafter, the sections were incubated with HRP‐labeled goat anti‐mouse (CD4, CD68, CD169, and TIA‐1) or anti‐rabbit (CD3) secondary antibodies (Nichirei Bioscience Inc.). Immunoreactivity was visualized using 3–3 *Diaminobenzidine Dab Substrate* Kits (Nichirei Bioscience Inc.). The negative control was isotype‐matched mouse or rabbit IgG (Agilent Technologies Inc.).

### 2.3 Histological analysis

**FIGURE 1:**
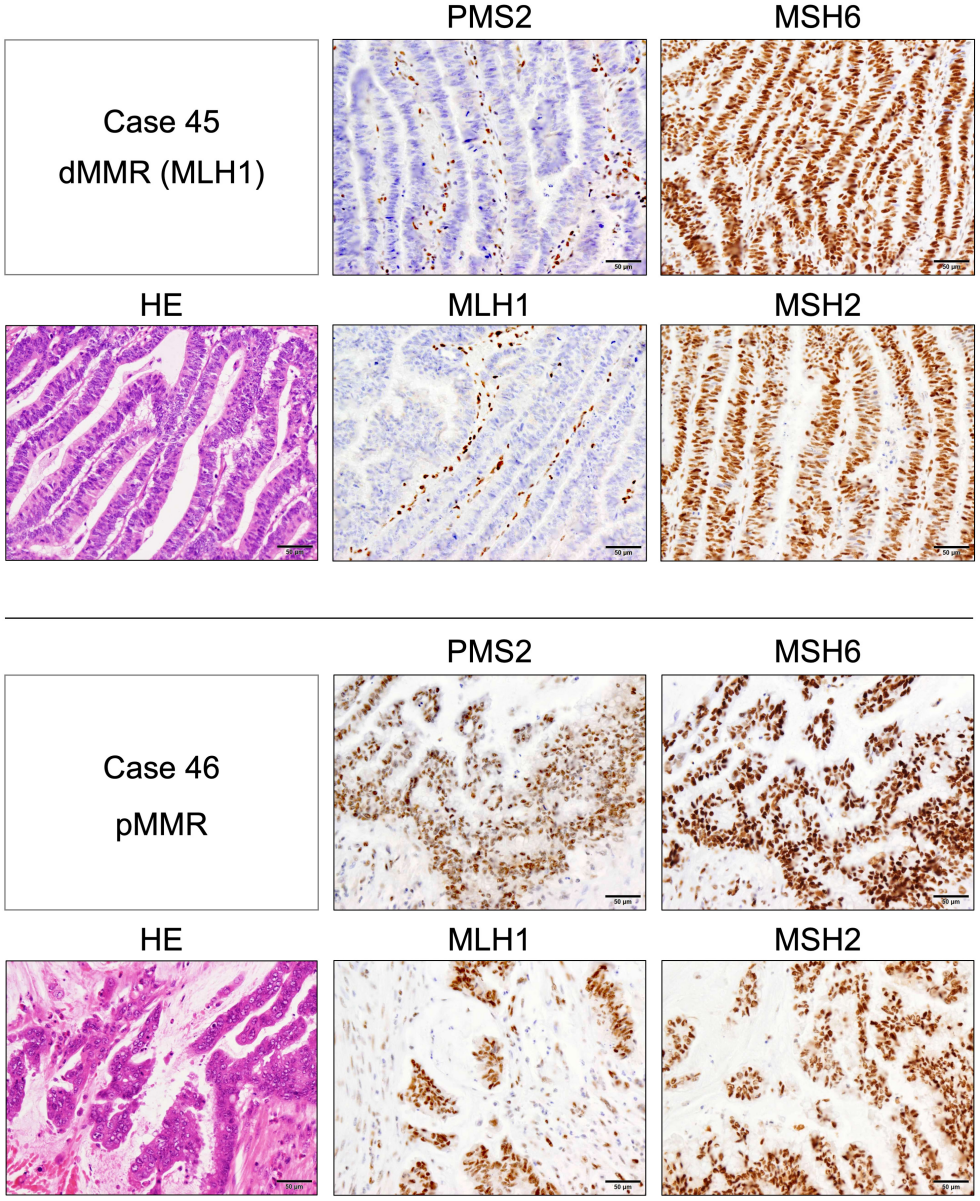
Immunohistochemical analysis of MMR proteins PMS2, MSH6, MLH1, and MSH2 in CRCs. Representative CRC specimens with dMMR and pMMR are shown with identity numbers and deficient proteins. The scale bars represent 50 μm.

Two pathologists (YS and KO), who were blinded to the sample information, histologically evaluated the sections. The negative expression of MMR protein was defined as the complete loss of nuclear staining in tumor cells, despite positive nuclear staining in some surrounding stromal cells or TILs (Figure 1). dMMR was defined as the negative expression of at least one MMR protein. pMMR was defined as the positive expression of all four MMR proteins. Figure S1 and Table S1 show the method used to identify responsible deficiencies in MMR proteins. The extent of CD3^+^, CD4^+^, CD8^+^, and TIA‐1^+^ T‐cell infiltration into the tumors and of CD68^+^ and CD169^+^ macrophages in the RLNs was evaluated in four independent fields by microscopy (magnification, 400×), and positive cells/mm^2^ were calculated as described previously.^38^ The CD169 ratio was determined by dividing the number of CD169^+^ cells by that of CD68^+^ cells. The expression of PD‐L1 in tumor cells was evaluated using tumor proportion score (TPS) and the combined positive score (CPS) based on the CDx criteria for pembrolizumab therapy.^25^, ^26^, ^27^, ^28^, ^29^, ^46^

### 2.4 Statistical analysis

Paired groups were analyzed using Prism (GraphPad Software Inc.). Individual values are expressed as means ± standard deviation (SD). Differences between two groups were analyzed using Mann–Whitney *U* tests. Values with *p* < 0.05 were considered statistically significant. The correlation between the number of CD8^+^ T cells in the tumors and the CD169 ratio was analyzed by simple linear regression. Individual values are represented as dot plots. The dashed line, equation, and *R* ^2^ value indicate the approximate line for all tumors and their correlation coefficients. The *p* value indicates the significance of the non‐zero slope. Survival curves were generated using SAS Studio (SAS Institute Inc.), and cumulative survival rates were compared between two groups using log‐rank and generalized Wilcoxon tests. The results were statistically analyzed using SAS Studio.

## 3 RESULTS

### 3.1 Immunostaining of MMR protein in 83 CRC samples revealed 9 dMMR cases

The MMR status of the samples was determined based on the presence or absence of immunostained MMR protein (Figure S1 and Table S1). Figure 1 shows typical staining images of each MMR status. Figure S2 shows that among nine dMMR tumors, three had PMS2 deficiency, four had MLH1 deficiency, and two had MSH2 deficiency, while none had MSH6 deficiency (Table 2). One poorly differentiated and two of the six mucinous carcinomas were dMMR tumors. Six of the nine dMMR tumors were localized in the right colon (ascending colon or cecum). The average and median ages of the patients with dMMR tumors were 58.9 and 54 years, respectively, and sex differences were not evident (female, *n* = 5; male, *n* = 4). Eight of the nine dMMR tumors were stage II, one was stage III and none were stages I or IV.

**TABLE 2:** MMR status of dMMR CRC cases.

Case no	Age (years)	Sex	Part	Histological subtype	Stage	MMR proteins				Deficiency
						PMS2	MSH6	MLH1	MSH2	
3	33	F	A	Tubular, well	II	+	−	+	−	MSH2
9	54	F	S	Tubular, well	II	−	+	+	+	PMS2
13	39	M	R	Tubular, mod	II	+	−	+	−	MSH2
27	76	F	A	por	IIIb	−	+	−	+	MLH1
31	71	F	A	Mucinous	II	−	+	+	+	PMS2
45	90	M	C	Tubular, well	II	−	+	−	+	MLH1
56	82	F	T	Tubular, well	II	(−)p	+	(−)p	+	MLH1
63	39	M	A	Tubular, mod	II	−	(−)p	+	(−)p	PMS2
70	46	M	A	Mucinous	II	−	+	−	+	MLH1

Abbreviations: (−)p, partially negative; A, ascending; C, cecum; mod, moderately differentiated; por, poorly differentiated; R, rectum; S, sigmoid; T, transverse; well, well differentiated.

### 3.2 Atypical deficiencies in MMR proteins in two of nine dMMR tumors

Deficiencies in the MMR proteins partially or overlapped in two of the nine dMMR tumors. Figure S3 shows that MLH1 was partially negative in case no. 56 and the area that was not stained coincided with a negative area in PMS2. We concluded that MLH1 was partially deficient.

In contrast, PMS2 was completely deficient (negative) in case no. 63 (Figure S2). However, MSH6 and MSH2 staining was partially negative in the same area (Figure S4), indicating that this case contained PMS2 completely deficient and MSH2 partially deficient.

### 3.3 MMR status was not associated with prognosis or TILs

**FIGURE 2:**
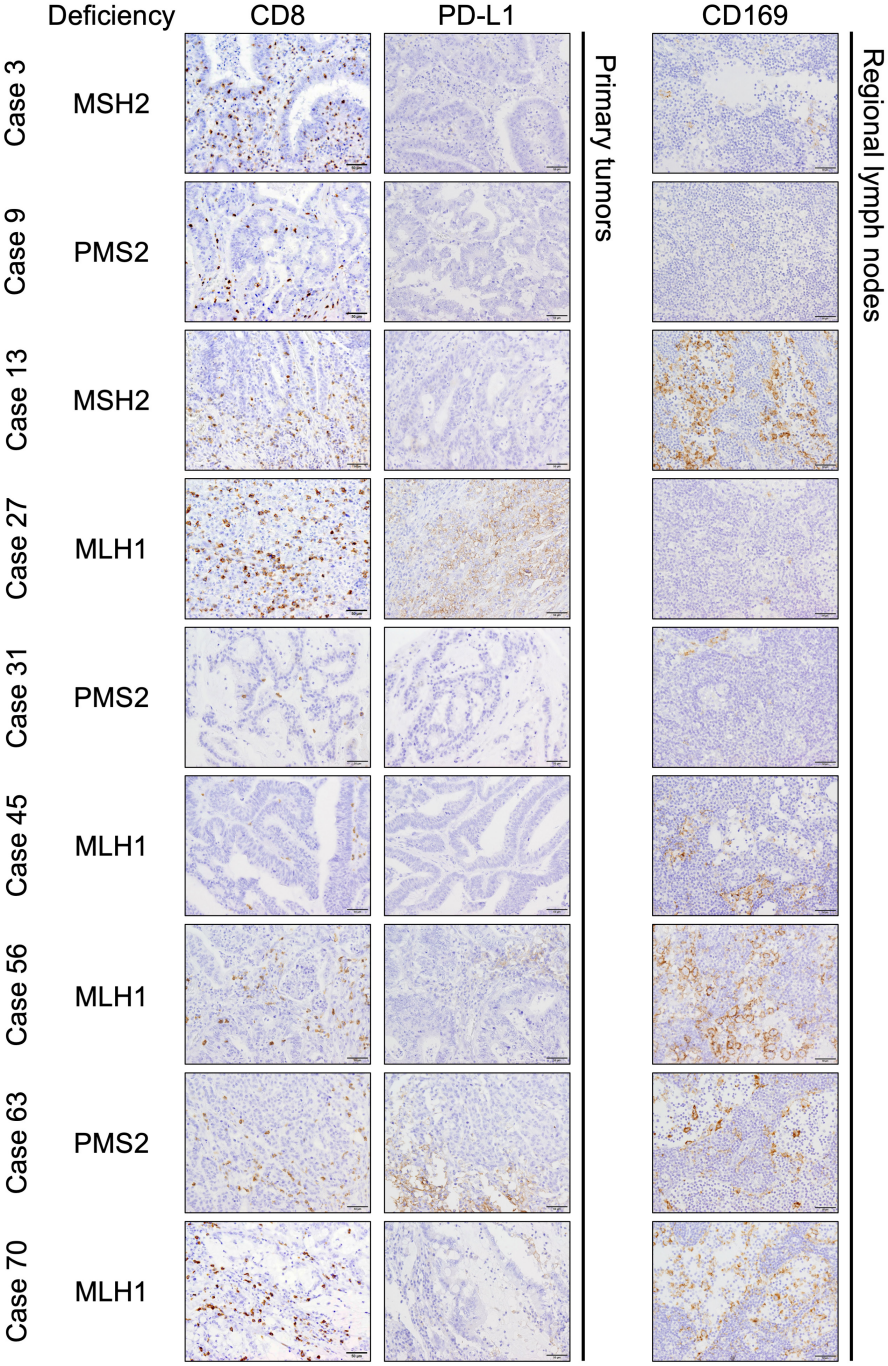
Immunohistochemical analysis of CD8 and PD‐L1 in CRCs and CD169 in RLNs in CRC with dMMR. All CRC specimens with dMMR are shown with identity numbers and mutated proteins. The scale bars represent 50 μm.

**FIGURE 3:**
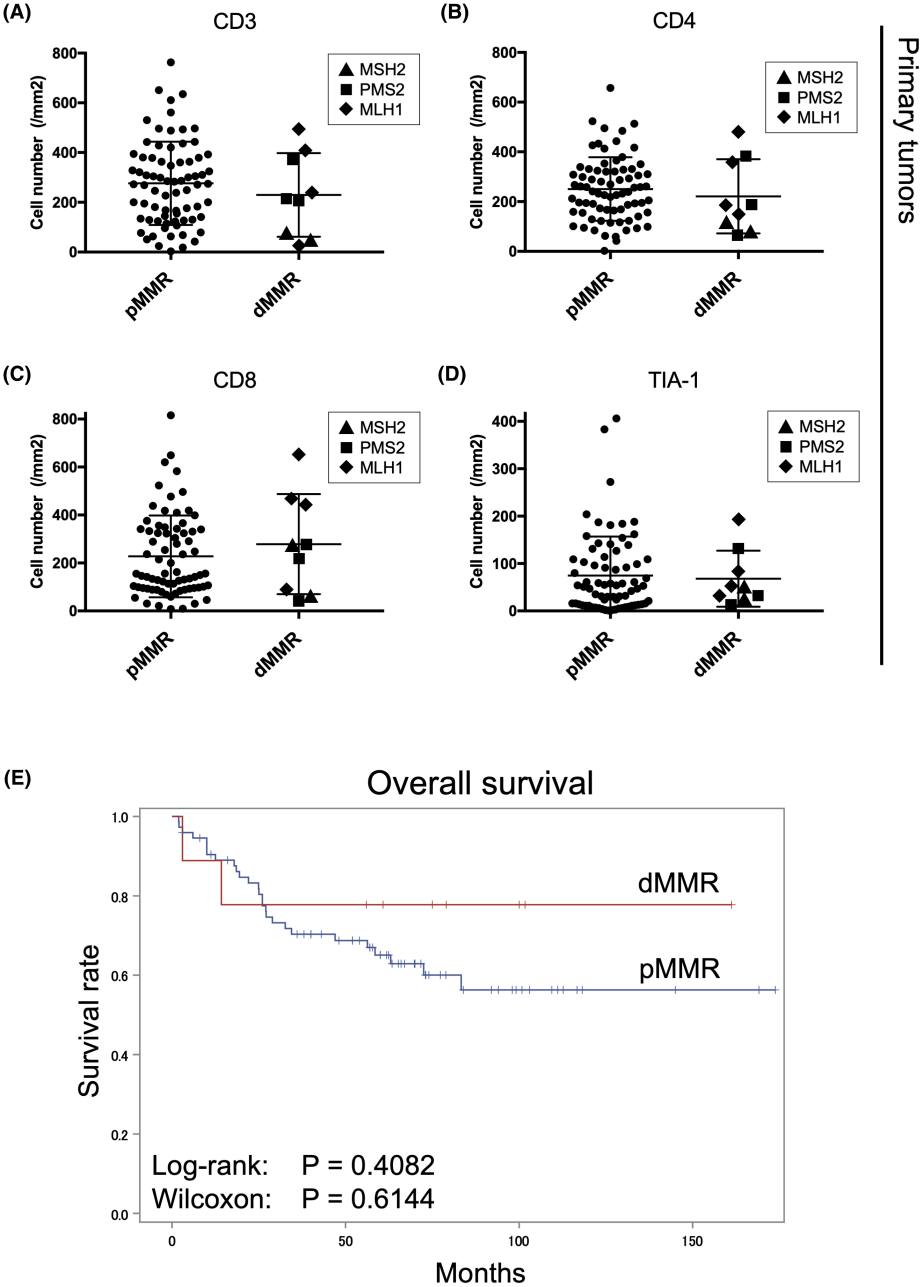
Comparison of amount of positive cells and prognosis in pMMR and dMMR CRCs. Dot plots of CD3^+^ (A), CD4^+^ (B), CD8^+^ (C), and TIA‐1^+^ (D) T cells in primary tumors. Triangles (▲), squares (■), and diamonds (◆) indicate cases with deficiency of MSH2, PMS2, and MLH1, respectively. (E) Kaplan–Meier overall survival curves for 83 patients with CRC according to MMR status.

The potentially high immunogenicity of dMMR/MSI‐H tumors results in abundant T‐cell infiltration. We compared T‐cell infiltration between tumors with pMMR and dMMR to clarify the relationship between dMMR and T‐cell‐mediated antitumor immunity. The abundance and distribution of TILs widely varied among tumors (Figure 2, Table 3, Figure S5, and Table S2). The numbers of CD3‐, CD4‐, CD8‐, and TIA‐1 positive cells did not significantly differ between pMMR and dMMR (Figure 3A–D), and dMMR did not significantly correlate with a good overall survival (OS) (*p* = 0.4082 log‐rank tests; *p* = 0.6144 Wilcoxon tests; Figure 3E). These results were similar when limited to stage II cases (Figure S6 and Table S3) (*p* = 0.7265 log‐rank tests; *p* = 0.8503 Wilcoxon tests; Figure S7).

**TABLE 3:** Immunohistological analyses of dMMR CRC cases.

dMMR case		Primary tumor						Regional lymph nodes		
Case no.	Deficiency	CD3^+^ cells (/mm^2^)	CD4^+^ cells (/mm^2^)	CD8^+^ cells (/mm^2^)	TIA‐1^+^ cells (/mm^2^)	PD‐L1^+^		CD68^+^ cells (/mm^2^)	CD169^+^ cells (/mm^2^)	CD169^+^/CD68^+^ ratio
						TPS **(%)**	CPS **(%)**			
3	MSH2	69	72	56	23	0	<1	161	25	0.155
9	PMS2	213	189	41	13	0	<1	191	5	0.026
13	MSH2	40	112	269	49	0	<1	287	366	1.275
27	MLH1	409	185	443	193	35	40	153	51	0.333
31	PMS2	205	65	217	33	0	<1	195	24	0.123
45	MLH1	26	148	90	33	0	<1	443	85	0.192
56	MLH1	239	358	468	83	0	1	328	399	1.216
63	PMS2	371	382	272	132	1	5	313	117	0.374
70	MLH1	494	480	652	53	0	3	315	202	0.641

Abbreviations: CPS, combined positive score; TPS, tumor proportion score.

### 3.4 CD169 in RLNs did not predict MMR status

**FIGURE 4:**
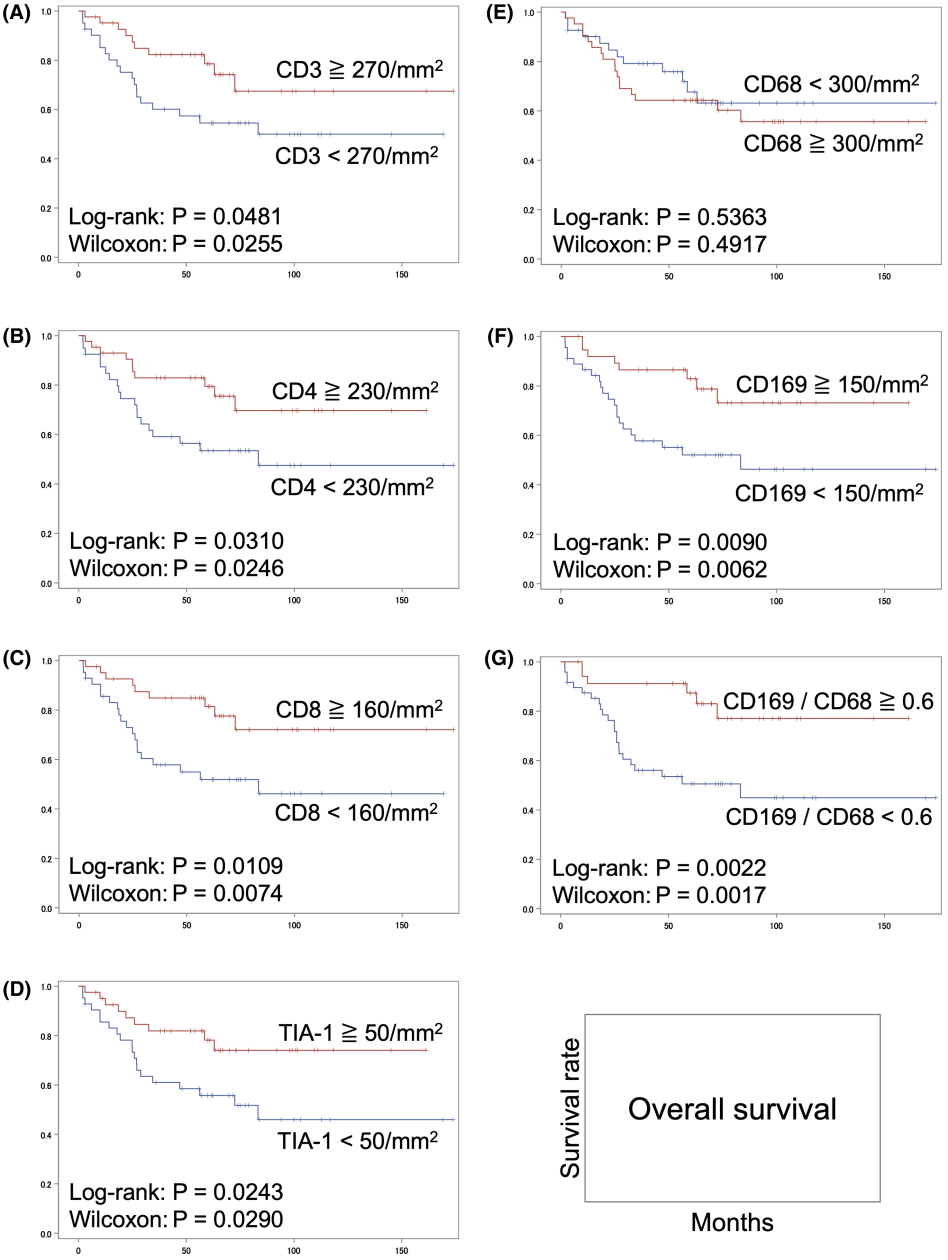
Kaplan–Meier overall survival curves for 83 patients with CRC according to numbers of positive cells in CRCs and the RLNs. Survival was associated with CD3^+^ (A), CD4^+^ (B), CD8^+^ (C), and TIA‐1^+^ (D) T cells in primary tumors, CD68^+^ (E) and CD169^+^ (F) macrophages in RLNs, and the ratio of CD169 to CD68 (G). The cutoff values were based on the median values.

**FIGURE 5:**
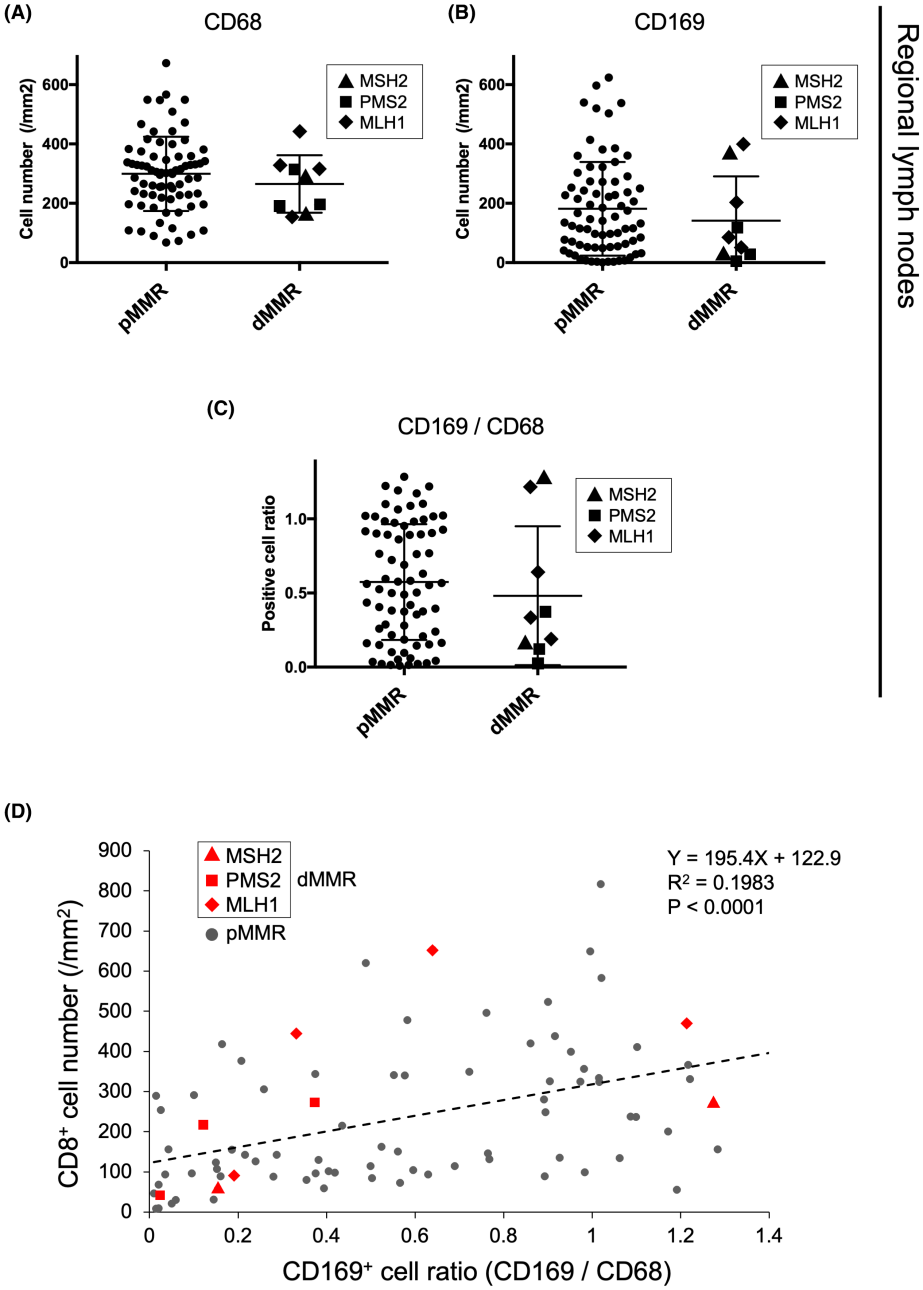
Comparison of numbers of sinus macrophages in RLNs of CRCs with pMMR and dMMR. Dot plots of CD68^+^ (A) and CD169^+^ (B) macrophages in RLNs and ratio of CD169 to CD68 (C). Triangles (▲), squares (■), and diamonds (◆) indicate cases with deficiency of MSH2, PMS2, and MLH1, respectively. Distribution of dMMR in dot plot of CD8^+^ T cell abundance in primary tumors and ratio of CD169^+^ to CD68^+^ macrophages in RLNs (D). Red triangles (▲), squares (■), and diamonds (◆) indicate cases with deficiency of MSH2, PMS2, and MLH1, respectively. The dashed line, equation, and R^2^ value indicate the approximate line for all tumors and their correlation coefficients. The P value indicates the significance of the non‐zero slope.

We previously showed that the numbers of CD169^+^ sinus macrophages in RLNs and that abundant CD8^+^ T‐cell infiltration significantly correlated with a favorable prognosis in the same patient population with CRC.^37^ In the present study, we updated clinical data and strengthened the statistical significance of these findings (Figure 4). To verify the ability of CD169 to predict MMR status, we compared the amount of sinus macrophages in the RLNs between pMMR and dMMR by immunostaining CD169 and the pan‐macrophage marker CD68. The number of cells positive for CD68 and CD169, and the ratio of CD169 to CD68 varied (Table 3 and Table S2), and did not significantly correlate with MMR status (Figure 5A–C). These results were similar when limited to stage II cases (Figure S8 and Table S3). In addition, although the ratios of CD169^+^ sinus macrophages in RLNs correlated positively with CD8^+^ T‐cell infiltration in CRC, a specific trend in the distribution of dMMR was not evident (Figure 5D). Thus, MMR status in patients with CRC could not be predicted based on CD169^+^ sinus macrophages in RLNs.

### 3.5 PD‐L1 expression was not associated with dMMR

ICIs, including PD‐(L)1 antibodies, confer clinical benefits upon patients with dMMR/MSI‐H CRC, but not with pMMR/MSS CRC.^31^ Treatment with ICIs notably requires only dMMR/MSI‐H status instead of PD‐L1 expression in CRC cells. We therefore quantified PD‐L1 expression in the nine CRC tumors with dMMR to confirm this criterion. Scores for PD‐L1 expression were remarkably high in one tumor (no. 27) and low in eight (TPS, 35% vs. ≤1%; CPS, 40% vs. ≤5%; Figure 2 and Table 3). These results indicated that MMR status and PD‐L1 expression are independent in CRC cells, which was similar to the findings of a comprehensive, systemic review.^12^

## 4 DISCUSSION

The estimated frequency of dMMR/MSI‐H among CRCs is 3%–15%. Next‐generation sequencing (*n* = 1395) and IHC detection of MMR protein (*n* = 925) have shown that MSI‐H and dMMR account for 5.7% and 6.5%, respectively, of CRCs.^47^ The frequency of MSI‐H CRC determined by PCR in Japan is 3.78% according to real‐world data.^48^ In the present study, IHC staining identified MMR in 9 (10.8%) of 83 resected CRCs (Figure S2; Tables 1 and 2). The higher frequency might have been due to the higher proportion of resected stage I and II tumors. Our dMMR identification is considered appropriate considering that two‐thirds of the tumors were localized to the right colon, none were stage IV, and three of seven poorly differentiated carcinomas were contained.

Cancers with dMMR/MSI‐H are highly antigenic and are likely to activate T‐cell‐mediated adaptive antitumor immunity. Infiltrative TILs are more abundant in MSI than in MSS CRC.^9^ However, A recent, comprehensive, systemic review did not find a significant correlation between MMR status and TILs.^12^ Furthermore, the prognosis of dMMR/MSI‐H CRC has not yet reached consensus. IHC analyses did not reveal any correlations between MMR status and TILs, or OS in our study cohort (Figure 3). We also did not find any correlations in 8 tumors with dMMR among 32 stage II CRC tumors (Figures S6 and S7). Our results also supported the notion that MMR status is not useful as an immune‐hot tumor predictor or prognostic factor for CRC.

However, the prognosis of CRC with high CD8^+^ TILs has long been considered favorable.^49^, ^50^, ^51^ The OS of the patients with CRC and high TILs was significantly longer (Figure 4A–D). This means that the prognosis is good when CRC becomes a hot tumor. To summarize, dMMR has the potential to be a hot tumor, but it is not the same. Without an event that induces antitumor immunity, even dMMR cannot become a hot tumor.

The expression of CD169 in sinus macrophages in RLNs monitors TILs and predicts a better prognosis.^37^, ^38^, ^40^, ^41^, ^42^, ^43^, ^44^, ^45^ The correlation between the high CD169^+^ sinus macrophages and longer OS was clear according to the updated clinical data (Figure 4F,G), and dMMR did not correlate with abundant CD169^+^ sinus macrophages in the RLNs (Figure 5B,C). Furthermore, the significant correlation between each histopathological factor and the prognosis was similar when restricted to pMMR cases (Figure S9). Since sinus macrophages in RLNs should have been exposed to more tumor neoantigens in dMMR than pMMR, we interpreted the data in Figure 5D to mean that the exposure of macrophages to neoantigens does not correlate with CD169 or TILs. Thus, the exposure to tumor neoantigens in RLNs does not seem to directly affect the establishment of subsequent T‐cell‐mediated adaptive antitumor immunity, and the high antigenicity of dMMR tumors is insufficient to activate host immunity.

A previous study found more PD‐L1 expression in MSI than in MSS CRC, and that most PD‐L1^+^ cells were tumor‐associated macrophages (TAMs) and not tumor cells.^9^ We immunostained PD‐L1 only in dMMR tumors due to financial constraints, and found one of nine tumors that was obviously positive for PD‐L1 and the others were only partially positive for PD‐L1 in TAMs, which agreed with the above results. However, the dMMR tumors did not tend to have high CPS. If the dMMR really correlates with PD‐L1 expression in CRCs, then including only dMMR to the CDx for PD‐1 antibodies and excluding PD‐L1 expression is not rational. Indeed, previous studies have shown that dMMR does not equal high PD‐L1 expression in CRC.^12^, ^47^ dMMR CRC predicts the therapeutic effect of PD‐1 antibodies, but probably not simply because dMMR is highly antigenic.

We found that CD169^+^ sinus macrophages in RLNs do not correlate with MMR status in patients with CRC. We also confirmed that CD169 predicts a better prognosis independently of MMR status. The clinical factors that produce high CD169 in RLNs and induce T‐cell‐mediated adaptive antitumor immunity remain unknown. However, the present findings indicated that dMMR CRC could not induce T‐cell‐mediated adaptive antitumor immunity despite having high tumor antigenicity. Type 1 IFNs (α and β) are closely associated with innate immunity and induce CD169 expression in macrophages in vitro.^37^, ^38^ The clinical conditions under which macrophage CD169 expression is controlled by type 1 IFN might be dictated by the host immune environment before tumorigenesis. To identify such conditions is critically important to improve the effect of ICIs. As the ICI applications expand, more surgically resected specimens will become available after treatment. We plan to determine how ICI treatment affects CD169 expression in sinus macrophages to resolve the above issues.

## AUTHOR CONTRIBUTIONS

**Yoichi Saito:** Conceptualization (lead); data curation (equal); formal analysis (lead); funding acquisition (equal); investigation (lead); methodology (lead); project administration (lead); validation (equal); visualization (lead); writing – original draft (lead); writing – review and editing (lead). **Yukio Fujiwara:** Conceptualization (supporting); data curation (supporting); formal analysis (supporting); funding acquisition (equal); investigation (equal); methodology (supporting); project administration (lead); supervision (equal); validation (equal); writing – original draft (supporting); writing – review and editing (supporting). **Yuji Miyamoto:** Data curation (equal); formal analysis (equal); investigation (supporting); resources (equal); supervision (supporting); validation (supporting). **Koji Ohnishi:** Conceptualization (supporting); data curation (equal); formal analysis (supporting); investigation (supporting); methodology (equal); project administration (supporting); supervision (supporting); validation (supporting). **Yuta Nakashima:** Funding acquisition (supporting); investigation (supporting); project administration (supporting); software (equal); supervision (supporting); validation (supporting); writing – original draft (supporting). **Yasuhiko Tabata:** Funding acquisition (supporting); investigation (supporting); project administration (supporting); supervision (equal); validation (supporting). **Hideo Baba:** Data curation (supporting); investigation (supporting); project administration (supporting); resources (supporting); supervision (equal); validation (supporting). **Yoshihiro Komohara:** Data curation (supporting); funding acquisition (supporting); project administration (supporting); supervision (supporting); validation (supporting); visualization (supporting).

## FUNDING INFORMATION

This study was supported in part by Grants‐in‐Aid for Scientific Research (18J01441, 19K07459, and 22K04845) from the Ministry of Education, Culture, Sports, Science, and Technology of Japan.

## CONFLICT OF INTEREST STATEMENT

The authors have no potential conflicts of interest to declare.

## ETHIC APPROVAL

The study protocols were approved by the Institutional Review Board at Kumamoto University Hospital (approval no. 1016).

## PATIENT CONSENT

All patients provided written informed consent to participate in the study.

## Supporting information


Figure S1.

Table S1.

Table S2.

Table S3.

## Data Availability

The datasets analyzed during the study are available from the corresponding author on reasonable request.
