# Supplementary material for: CD169 + sinus macrophages in regional lymph nodes do not predict mismatch‐repair status of patients with colorectal cancer
Source: Cancer Med. 2023 Feb 27;12(9):10199–211. doi: 10.1002/cam4.5747 (PMC10225197; doi:10.1002/cam4.5747)
Supplement: Supplementary file 1 — Figure S1. [file CAM4-12-10199-s001.docx]

**Supplemental figure legends:**

Supplemental figure S1. Immunostaining procedure for detecting dMMR cases. For the first staining, all primary tumor samples were stained with the MMR proteins (PMS2 and MSH6) and T cell markers (CD3, CD4, CD8, TIA-1). All regional lymph node samples were also stained with macrophage markers (CD68 and CD169). There were 74 pMMR cases that were PMS2^+^ and MSH6^+^ and 7 dMMR cases that were PMS2^-^ and/or MSH6^-^. For the second staining, seven dMMR primary tumor samples were stained with remaining two MMR proteins (MLH1 and MSH2) and PD-L1.

Supplemental figure S2. Immunohistochemical analysis of MMR proteins PMS2, MSH6, MLH1, and MSH2 in CRCs. All CRC specimens with dMMR and representative pMMR are shown with identity numbers and deficient proteins. The scale bars represent 50 μm.

Supplemental figure S3. Immunohistochemical analysis of PMS2 and MLH1 in the case 56, MLH1 partially mutant. The red dot lines indicate border of positive and negative staining. The scale bars represent 200 μm.

Supplemental figure S4. Immunohistochemical analysis of MSH6 and MSH2 in the case 63, PMS2 mutant with MSH2 partially mutant. The right panels are higher magnification images of the squared area in the left panels. The red dot lines indicate border of positive and negative staining. The scale bars of the left and right represent 200 μm and 100 μm, respectively.

Supplemental figure S5. Immunohistochemical analysis of CD3, CD4, CD8, and TIA-1 in serial sections of primary tumors. The scale bars represent 20 μm.

Supplemental figure S6. Comparison of numbers of positive cells in stage II pMMR and dMMR CRCs. Dot plots of CD3^+^ (A), CD4^+^ (B), CD8^+^ (C), and TIA-1^+^ (D) T cells in primary tumors. Triangles (▲), squares (■), and diamonds (◆) indicate cases with deficiency of MSH2, PMS2, and MLH1, respectively.

Supplemental figure S7. Overall Kaplan-Meier survival curves for the 32 patients with stage II CRC as related to MMR status.

Supplemental figure S8. Comparison of numbers of sinus macrophages in RLNs of Stage II CRCs with pMMR and dMMR. Dot plots of CD68^+^ (A) and CD169^+^ (B) macrophages in RLNs and ratio of CD169 to CD68 (C). Triangles (▲), squares (■), and diamonds (◆) indicate cases with deficiency of MSH2, PMS2, and MLH1, respectively.

Supplemental figure S9. Kaplan-Meier overall survival curves for 74 patients with pMMR CRC according to numbers of positive cells in CRCs and the RLNs. Survival was associated with CD3^+^ (A), CD4^+^ (B), CD8^+^ (C), and TIA-1^+^ (D) T cells in primary tumors, CD68^+^ (E) and CD169^+^ (F) macrophages in RLNs, and the ratio of CD169 to CD68 (G). The cutoff values were based on the median values.
