# Supplementary material for: CD169 + sinus macrophages in regional lymph nodes do not predict mismatch‐repair status of patients with colorectal cancer
Source: Cancer Med. 2023 Feb 27;12(9):10199–211. doi: 10.1002/cam4.5747 (PMC10225197; doi:10.1002/cam4.5747)
Supplement: Supplementary file 2 — Table S1. [file CAM4-12-10199-s004.docx]

| **Table S1.** Determination of deficiency by immunostaining pattern of MMR proteins | | | | | |
| --- | --- | --- | --- | --- | --- |
| **MMR status** | **Immunostaining of MMR proteins** | | | | **Deficiency** |
|  | **PMS2** | **MSH6** | **MLH1** | **MSH2** |  |
| pMMR | + | + | (Not done) | | none |
| dMMR | - | + | + | + | PMS2 |
|  | + | - | + | + | MSH6 |
|  | - | + | - | + | MLH1 |
|  | + | - | + | - | MSH2 |
