# Supplementary material for: CD169 + sinus macrophages in regional lymph nodes do not predict mismatch‐repair status of patients with colorectal cancer
Source: Cancer Med. 2023 Feb 27;12(9):10199–211. doi: 10.1002/cam4.5747 (PMC10225197; doi:10.1002/cam4.5747)
Supplement: Supplementary file 3 — Table S2. [file CAM4-12-10199-s003.docx]

| **Table S2.** Imunnohistological charactaristics | | | | | |
| --- | --- | --- | --- | --- | --- |
|  |  |  | **Possitive cell number (/mm2)** | | |
| **MMR status** | | | **pMMR** |  | **dMMR** |
|  |  |  | ***n* = 74 (89%)** |  | ***n* = 9 (11%)** |
| **Primary tumor** | | |  |  |  |
|  | **CD3** | |  |  |  |
|  |  | Median (range) | 273 (3-763) |  | 213 (26-494) |
|  |  | Mean | 276 |  | 230 |
|  | **CD4** | |  |  |  |
|  |  | Median (range) | 240 (2-657) |  | 185 (65-480) |
|  |  | Mean | 250.6 |  | 221.2 |
|  | **CD8** | |  |  |  |
|  |  | Median (range) | 156 (8-816) |  | 269 (41-652) |
|  |  | Mean | 228 |  | 279 |
|  | **TIA-1** | |  |  |  |
|  |  | Median (range) | 52 (1-406) |  | 49 (13-193) |
|  |  | Mean | 74.6 |  | 68 |
|  |  |  |  |  |  |
| **Regional lymph node** | | |  |  |  |
|  | **CD68** | |  |  |  |
|  |  | Median (range) | 302.5 (68-673) |  | 287 (153-443) |
|  |  | Mean | 299.3 |  | 265.1 |
|  | **CD169** | |  |  |  |
|  |  | Median (range) | 138 (1-624) |  | 85 (5-399) |
|  |  | Mean | 181.6 |  | 141.6 |
|  |  |  |  |  |  |
