# Supplementary material for: CD169 + sinus macrophages in regional lymph nodes do not predict mismatch‐repair status of patients with colorectal cancer
Source: Cancer Med. 2023 Feb 27;12(9):10199–211. doi: 10.1002/cam4.5747 (PMC10225197; doi:10.1002/cam4.5747)
Supplement: Supplementary file 4 — Table S3. [file CAM4-12-10199-s002.docx]

| **Table S3.** Imunnohistological charactaristics (Stage II) | | | | | |
| --- | --- | --- | --- | --- | --- |
|  |  |  | **Possitive cell number (/mm2)** | | |
| **MMR status** | | | **pMMR** |  | **dMMR** |
|  |  |  | ***n* = 24 (75%)** |  | ***n* = 8 (25%)** |
| **Primary tumor** | | |  |  |  |
|  | **CD3** | |  |  |  |
|  |  | Median (range) | 222 (24-493) |  | 209 (26-494) |
|  |  | Mean | 228 |  | 207 |
|  | **CD4** | |  |  |  |
|  |  | Median (range) | 208.5 (59-495) |  | 168.5 (65-480) |
|  |  | Mean | 227 |  | 225.8 |
|  | **CD8** | |  |  |  |
|  |  | Median (range) | 156 (31-816) |  | 243 (41-652) |
|  |  | Mean | 208 |  | 258 |
|  | **TIA-1** | |  |  |  |
|  |  | Median (range) | 27 (2-188) |  | 41 (13-132) |
|  |  | Mean | 54.9 |  | 52.4 |
|  |  |  |  |  |  |
| **Regional lymph node** | | |  |  |  |
|  | **CD68** | |  |  |  |
|  |  | Median (range) | 260.5 (94-567) |  | 300 (161-443) |
|  |  | Mean | 284.1 |  | 279.1 |
|  | **CD169** | |  |  |  |
|  |  | Median (range) | 108 (3-540) |  | 101 (5-399) |
|  |  | Mean | 160.9 |  | 152.9 |
|  |  |  |  |  |  |
